# Interval training has more negative effects on sleep in adolescent speed skaters: a randomized cross controlled trial

**DOI:** 10.3389/fspor.2024.1367190

**Published:** 2024-04-16

**Authors:** Zhenxing Kong, Xinhua Wei, Meng Shen, Yue Cheng, Junpeng Feng

**Affiliations:** ^1^ Key Laboratory of Exercise and Physical Fitness of the Ministry of Education, Beijing Sport University, Beijing, China; ^2^ Zhejiang Qiangnao Technology Co., Ltd, Hangzhou, Zhejiang, China; ^3^ Ice Sports Management Center of Jilin Provincial Sports Bureau, Changchun, Jilin, China

**Keywords:** speed skating, sleep, Wingate Test, interval training, high-intensity exercise

## Abstract

**Objective:**

Sleep is an essential component of athletic performance and recovery. This study aimed to investigate the effects of different types of high-intensity exercise on sleep parameters in adolescent speed skaters.

**Methods:**

Eighteen male adolescent speed skaters underwent aerobic capacity testing, Wingate testing, and interval training in a randomized crossover design to assess strength output, heart rate, and blood lactate levels during exercise. Sleep quality after each type of exercise was evaluated using the Firstbeat Bodyguard 3 monitor.

**Results:**

The results showed that Wingate testing and interval training led to decreased sleep duration, increased duration of stress, decreased RMSSD, and increased LF/HF ratio (*p* < 0.01). Conversely, aerobic capacity testing did not significantly affect sleep (*p* > 0.05). The impact of interval training on sleep parameters was more significant compared to aerobic capacity testing (*p* < 0.01) and Wingate testing (*p* < 0.01).

**Conclusion:**

High-intensity anaerobic exercise has a profound impact on athletes’ sleep, primarily resulting in decreased sleep duration, increased stress duration, decreased RMSSD, and increased LF/HF ratio.

## 1 Introduction

Sleep is an essential component of human physiological activities, playing a crucial role in the body's recovery, maintenance of immune function, and regulation of psychological well-being (1–4). For athletes, good sleep is not only related to the body's rehabilitation and repair but also directly influences their training outcomes and athletic performance (5–7).

Moderate exercise is considered a crucial means to maintain overall health and promote sleep (8). Research indicates that regular aerobic exercises, such as jogging and swimming, can enhance sleep quality, shorten the time it takes to fall asleep, and reduce the frequency of nighttime awakenings (9). This is mainly attributed to the fact that exercise can elevate body temperature, followed by a gradual decline in temperature after exercise. This temperature change stimulates the sensitivity of the biological clock to temperature variations, making it easier for individuals to fall asleep. Additionally, exercise promotes the secretion of neurotransmitters such as endorphins, inducing a calming and pleasant sensation, which helps alleviate tension, reduce anxiety levels, and consequently enhance sleep quality (8, 10, 11).

The impact of different intensities of exercise on sleep may vary. Due to the high-intensity and high-frequency training and competition activities, athletes experience a more complex and profound effects on their sleep (12, 13). With the continuous deepening of research on the relationship between exercise and sleep, increasing evidence indicates that exercise significantly influences the sleep of athletes. Studies have found that prolonged high-intensity training and excessive sports competition may disrupt the athletes' circadian rhythm, making it difficult for them to fall asleep at night and causing daytime fatigue (14–16). Especially on the night before a competition, athletes may face emotions such as tension and excitement, increasing the risk of insomnia (17–19). Additionally, athletes may be affected in their ability to fall asleep and sleep quality due to issues such as bodily pain and muscle soreness during the training process, consequently affecting their training and competitive performance (18, 20).

In order to understand the effects of different types of training on athletes' sleep, this paper aims to explore the impact of various types of high-intensity exercise on sleep parameters in adolescent speed skaters. This provides a scientific basis for optimizing athletes' periodic training schedules and sleep management.

## 2 Participants and methods

### 2.1 Participants

This study recruited 18 male youth professional speed skaters from provincial teams in northeastern China. All athletic levels were Level 1 or above. The athletes' age, heights, and weights are shown in Table 1.

**Table 1:** Basic profiles for elite male athletes (*n* = 18).

Age (year)	Height (cm)	Body weight (kg)
16.19 ± 1.23	174.42 ± 3.52	62.82 ± 5.36

Their best results for each distance were from the website https://www.speedskatingresults.com/.

All athletes were informed of the study procedures before testing and signed a written informed consent that they confirmed to be free of heavy training, competition, and injury before performing each test.

### 2.2 Design

All subjects completed 3 types of exercise including Aerobic Capacity Test, Wingate test, and Interval Training, one week between each type of exercise. All participants were randomly assigned three tests in order. All participants refrained from engaging in moderate to high-intensity exercise during the experiment and maintained normal sleep-wake schedules. The diet on the day preceding each of the three experiments was standardized.

Relative maximum power, relative average power, and heart rate were recorded during each type of exercise, fingertip blood was collected at the immediate end of exercise for blood lactate concentration analysis using an EKF portable blood lactate meter(EKF Lacate-Scout, EKF, Germany) and subjects were asked about their level of fatigue by Borg (6–20) Rating of Perceived Exertion (RPE) scale.

#### 2.2.1 Aerobic capacity test

Test Method: Subjects wore portable gas metabolic analyzer Cortex Metamax 3B R2 for power bike incremental load experiment, and the changes of heart rate and oxygen uptake values of subjects were monitored and recorded throughout the test. VO2max, heart rate, and anaerobic threshold were recorded during the test.

Incremental Load Scheme: The incremental load scheme is shown in Table 2 (21).

**Table 2:** The cycling incremental load scheme.

Level	Power(W)	Duration(min)	Rpm(r/min)
Rest	0	1	0
Level 1	0	1	70
Level 2	150	3	70
Level 3	200	3	70
Level 4	250	3	70
Level 5	300	3	70
…	…	3	70
Recovery	50	3	/

Termination Criteria: (1) The athlete feels exhausted and is unable to continue the exercise. (2) The athlete is below 10% of the designated speed for 30 s continuously. (3) A plateau in oxygen uptake as the load rises.

#### 2.2.2 Wingate test

The Wingate test was conducted using a power cycle (Monark 824E, Sweden) for athletes. Prior to the test, athletes were required to engage in a 25–30 min warm-up activity, and the body mass of each athlete was measured on-site. The resistance factor for each athlete was set at 7.5% of their body mass. The height of the power cycle seat was adjusted to be level with the greater trochanter of the femur. At the beginning of the test, the tester raised the resistance basket, and the researchers counted down from 5 to 1. Upon the command “start,” the athlete assumed a standing position and pedaled with maximum effort. Simultaneously, the resistance basket was released, initiating data collection. Throughout the test, the tester continuously encouraged the athletes to maintain the maximum exercise time for 30 s. Additionally, athletes were instructed not to lift their hips off the bicycle seat during the test (22).

#### 2.2.3 Interval training

Interval training was performed using a Wattbike (Wattbike Pro, UK). The training program required the athlete to complete five 150-second all-out workouts with 240 s of intervals between each repetition.

### 2.3 Sleep monitoring

The Firstbeat Bodyguard 3 sleep monitor (Firstbeat Bodyguard 3, Finland)was used to monitor the sleep quality of the subjects on the nights without any training and following each type of exercise. 7 parameters were selected, including: Sleep duration, Relaxation Time, Stress time, Average HR (Average Heart Rate), Average RespR (Average Respiratory Rate), as well as heart rate variability indicators RMSSD, LF/HF (23).

### 2.4 Statistical analyses

IBM SPSS Statistics (version 22.0; IBM Corp, Armonk, NY) performed the statistical analyses. All data obtained from the experiments were presented as mean ± standard deviation. Normality and homogeneity of variance tests were conducted on all data before analysis. Paired *t*-tests were used to compare changes in sleep parameters after different types of exercises within the same subjects, while independent samples *t*-tests were employed to analyze differences in training performance and sleep parameters among different subjects after different types of training. When *p* < 0.05, the statistical differences were significant, and when *p* < 0.01, the statis-tical differences were highly significant.

## 3 Results

### 3.1 Performance of athletes during different types of exercise

The data in Table 3 indicates certain differences in power output among the three types of exercises. The maximum and average power achieved in the Wingate Test are significantly higher than those in the Aerobic Capacity Test (*p* < 0.01) and Interval Training (*p* < 0.01). Among the three types of training, post-Interval Training resulted in the highest blood lactate concentration, significantly higher than the Aerobic Capacity Testm (*p* < 0.01) and Wingate Test (*p* < 0.01). There was no significant difference in the maximum heart rate of the subjects among the three types of training (*p* > 0.05), all exceeding 90% of the HRmax. Additionally, there was no significant difference in the Rating of Perceived Exertion (RPE) among the three types of training (*p* > 0.05).

**Table 3:** Performance of athletes during different types of exercise.

	Aerobic Capacity Test	Wingate Test	Interval training
Maximum power (W/kg)	4.16 ± 0.29	16.76 ± 3.26**	6.76 ± 0.41^##^
Average power (W/kg)	–	9.70 ± 0.79	5.74 ± 0.26^##^
Maximum heart rate (bpm)	193.50 ± 5.54	190.60 ± 3.66	190.90 ± 4.17
Maximal blood lactate (mmol/L)	12.24 ± 1.63	15.97 ± 1.58**	17.68 ± 0.94**^,^^##^
RPE	17.30 ± 0.95	18.20 ± 1.23	18.40 ± 0.79

*p < 0.05, **p < 0.01 when compared with Aerobic Capacity Test; ^#^*p* < 0.05, ^##^*p* < 0.01 when compared with Wingate Test.

### 3.2 Sleep parameters after different types of exercise

The data in Table 4 indicates that there are certain differences in the effects of the three types of exercises on sleep. After the Aerobic Capacity Test and Wingate Test, sleep duration, relaxation time, stress time, and the percentage of stress time all showed a decreasing trend compared to the no-training condition, but the differences were not statistically significant (*p* > 0.05). However, after Interval Training, sleep duration, relaxation time, stress time, and the percentage of stress time all significantly decreased (*p* < 0.05) compared to the no-training condition. The data showed that there was a significant increase in HF/LF and a significant increase in RMSSD of HRV during sleep compared to no-training after performing the Wingate Test (*p* < 0.05) and Interval Training (*p* < 0.01). Interval training had a more profound effect on sleep parameters than Aerobic Capacity Test (*p* < 0.01) and the Wingate Test (*p* < 0.01).

**Table 4:** Sleep parameters after different types of exercise.

	Without any exercise	Aerobic Capacity Test	Wingate Test	Interval training
Sleep duration (min)	478.50 ± 42.84	468.40 ± 23.27	455.70 ± 42.06	418.60 ± 44.85^&&^^,^*^,^^##^
Relaxation time (min)	422.10 ± 59.38	419.10 ± 30.50	380.40 ± 59.95	329.20 ± 54.54^&&^^,^**^,^^##^
Stress time (min)	50.00 ± 42.77	40.50 ± 16.30	63.50 ± 23.89*	84.20 ± 20.69^&^^,^**^,^^##^
Percentage of stress time to sleep duration	10.52% ± 8.96%	8.68% ± 3.49%	14.22% ± 5.91%*	20.47% ± 6.16%^&^^,^^##^
Average HR (bpm)	52.70 ± 2.94	51.10 ± 2.13	51.50 ± 0.97	51.80 ± 1.48
Average RespR (L/min)	15.55 ± 1.24	14.78 ± 0.99	14.89 ± 1.11	14.87 ± 1.25
RMSSD (ms)	76.65 ± 20.65	76.77 ± 14.66	65.93 ± 7.15*	57.43 ± 9.88^&^^,^**^,^^##^
LF/HF	1.76 ± 0.33	1.85 ± 0.16	2.06 ± 0.12^&^	2.42 ± 0.21^&&^^,^**^,^^##^

^&^*p* < 0.05, ^&&^*p* < 0.01 when compared with Without Any Exercise; *p < 0.05, **p < 0.01 when compared with Aerobic Capacity Test; ^#^*p* < 0.05, ^##^*p* < 0.01 when compared with Wingate Test.

## 4 Discussion

This study explores the influence of different types of exercise on the sleep of youth speed skaters. The three types of exercises employed in this study all fall under high-intensity activities, but they belong to high-intensity aerobic exercise and anaerobic exercise, resulting in variations in their effects on sleep. This study found that high-intensity anaerobic exercise has a more profound effects on sleep, especially concerning stress duration and heart rate variability.

Factors influencing athletes' sleep can be categorized into exercise-related and non-exercise-related factors (2, 24). Exercise-related factors include high training loads, nighttime competitions, long-distance travel, pre-competition nights, morning training, and unfamiliar training environments, among others (6). Compared to the general population, athletes are more significantly affected by training, competitions, and travel.

Halson et al. (6)conducted a sleep survey on 479 athletes from various events participating in the Tokyo Olympics (371 female athletes, 108 male athletes). The results revealed that more than half (52%) of the athletes experienced poor sleep quality. The prevalence of sleep issues among elite athletes has garnered attention. Addressing athletes’ sleep problems, Sargent et al. (19)approached the question of “how much sleep one should get” and found that elite male and female athletes perceived a need for 8.3 h of sleep for adequate rest. However, 71% of athletes were unable to meet this requirement.

Athletes must adhere strictly to coaches’ training plans to ensure optimal performance in major competitions (25). However, high-level athletes are constantly at risk of overtraining, which can lead to sleep issues. For example, in one study, athletes in the overtraining group had less sleep duration compared to the normal training group and acute fatigue group (26–28).

The different intensities and types of exercise lead to varied effects on sleep (29). Generally, low-intensity exercises such as jogging and yoga are believed to have the most positive effects on athletes' sleep quality (30). Research indicates that after engaging in low-intensity exercise, athletes experience relatively shorter sleep onset times, fewer nighttime awakenings, and an improvement in the proportion of deep sleep. Low-intensity exercise helps alleviate physical fatigue, reduce excitatory hormone levels, and facilitates athletes in entering a restful state more easily.

Studies indicate that sleep after high-intensity exercise typically exhibits characteristics such as prolonged sleep onset, reduced deep sleep, and increased nighttime awakenings (13, 31, 32). This may be linked to physiological activation, elevated body temperature, and changes in hormone levels post-exercise. However, it is important to note that individuals vary in their adaptability to high-intensity exercise, and some athletes may find it easier to adapt while maintaining a normal sleep pattern. High-intensity exercise significantly influences the hormone levels of athletes. Exercise induces the release of excitatory hormones such as adrenaline, noradrenaline, and growth hormone, which play essential roles in alertness and stress responses. However, if high-intensity exercise is performed before bedtime, it may lead to excessively elevated hormone levels, making it challenging for athletes to quickly enter a state of rest, effecting their sleep onset time and depth (16, 31, 32).

The mechanisms of high-intensity exercise on athlete sleep primarily involve physiological activation, elevated body temperature, hormonal fluctuations, muscle fatigue, and psychological stress (3, 33, 7). The physiological activation induced by high-intensity exercise may serve as a key factor influencing sleep. High-intensity exercise stimulates the release of excitatory hormones such as adrenaline and noradrenaline, increasing the athlete's alertness and arousal, thereby leading to increased difficulty in falling asleep (34). Another mechanism that may affect sleep is the elevation of body temperature following high-intensity exercise. Post-exercise, body temperature rises and remains elevated for a period, while sleep typically occurs in an environment where temperature gradually decreases. The divergent trends in body temperature may disrupt the regulation of the circadian rhythm, effecting sleep quality (35).

Muscle fatigue and the sensation of soreness induced by high-intensity exercise may influence the athlete's sleep onset and sleep quality (1). This discomfort may lead to an uncomfortable sleeping position, thereby affecting uninterrupted sleep throughout the night. High-intensity exercise is often accompanied by psychological stress. This stress may predispose athletes to the risk of insomnia, effecting their overall sleep performance. The relationship between psychological stress and insomnia is bidirectional, wherein psychological stress may be a cause of insomnia, and insomnia itself may contribute to increased psychological stress in athletes (33, 36, 37).

Significant fluctuations in hormone levels are also a focal point of research. Following exercise, particularly high-intensity anaerobic exercise, the release of excitatory hormones may heighten the athlete's alertness. This hormonal fluctuation can result in prolonged sleep onset and reduced deep sleep. Interestingly, different types of anaerobic exercise may have varying effects on hormone levels, necessitating further in-depth research to elucidate this relationship (38).

This study found that in high-intensity exercise, different types of sports also have varying effects on sleep (39). Based on the results of this study, we speculate that the prolonged accumulation of lactate and prolonged excitation of the nervous system caused by interval training may be crucial factors contributing to this difference, with the sustained activation of the nervous system playing a potentially more significant role. The differences in the effects on sleep also necessitate high-level athletes to engage in thorough cool-down activities and proactive recovery after high-intensity anaerobic training, thereby minimizing the effects on sleep.

## 5 Conclusions

High-intensity anaerobic exercise has a more profound effect on athlete sleep, primarily resulting in decreased sleep duration, increased stress duration, decreased RMSSD, and elevated LF/HF.

## Data Availability

The raw data supporting the conclusions of this article will be made available by the authors, without undue reservation.

## References

[1] Chennaoui M Vanneau T Trignol A Arnal P Gomez-Merino D Baudot C How does sleep help recovery from exercise-induced muscle injuries? J Sci Med Sport. (2021) 24(10):982–7. 10.1016/j.jsams.2021.05.007 34074604

[2] Dolezal BA Neufeld EV Boland DM Martin JL Cooper CB. Interrelationship between sleep and exercise: a systematic review. Adv Prev Med. (2017) 2017:1–14. 10.1155/2017/1364387 PMC5385214 28458924

[3] Krueger JM Rector DM Roy S Van Dongen HPA Belenky G Panksepp J. Sleep as a fundamental property of neuronal assemblies. Nat Rev Neurosci. (2008) 9(12):910–9. 10.1038/nrn2521 18985047 PMC2586424

[4] Memon AR Gupta CC Crowther ME Ferguson SA Tuckwell GA Vincent GE. Sleep and physical activity in university students: a systematic review and meta-analysis. Sleep Med Rev. (2021) 58:101482. 10.1016/j.smrv.2021.101482 33864990

[5] Clemente FM Afonso J Costa J Oliveira R Pino-Ortega J Rico-Gonzalez M. Relationships between sleep, athletic and match performance, training load, and injuries: a systematic review of soccer players. Healthcare. (2021) 9(7). 10.3390/healthcare9070808 PMC8305909 34206948

[6] Halson SL Johnston RD Appaneal RN Rogers MA Toohey LA Drew MK Sleep quality in elite athletes: normative values, reliability and understanding contributors to poor sleep. Sports Med. (2022) 52(2):417–26. 10.1007/s40279-021-01555-1 34554425

[7] Walsh NP Halson SL Sargent C Roach GD Nedelec M Gupta L Sleep and the athlete: narrative review and 2021 expert consensus recommendations. Br J Sports Med. (2020). 10.1136/bjsports-2020-102025 33144349

[8] Kalak N Gerber M Kirov R Mikoteit T Yordanova J Pühse U Daily morning running for 3 weeks improved sleep and psychological functioning in healthy adolescents compared with controls. J Adolesc Health. (2012) 51(6):615–22. 10.1016/j.jadohealth.2012.02.020 23174473

[9] Jurado-Fasoli L De la O A Molina-Hidalgo C Migueles JH Castillo MJ Amaro-Gahete FJ Exercise training improves sleep quality: a randomized controlled trial. Eur. J. Clin. Invest. (2020) 50(3). 10.1111/eci.13202 31989592

[10] Leduc C Tee J Weakley J Ramirez C Jones B. The quality, quantity, and intraindividual variability of sleep among students and student-athletes. Sports Health: A Multidisciplinary Approach. (2020) 12(1):43–50. 10.1177/1941738119887966 PMC6931182 31730421

[11] Myllymäki T Rusko H Syväoja H Juuti T Kinnunen M Kyröläinen H. Effects of exercise intensity and duration on nocturnal heart rate variability and sleep quality. Eur J Appl Physiol. (2012) 112(3):801–9. 10.1007/s00421-011-2034-9 21667290

[12] Alarcón-Gómez J Chulvi-Medrano I Martin-Rivera F Calatayud J. Effect of high-intensity interval training on quality of life, sleep quality, exercise motivation and enjoyment in sedentary people with type 1 diabetes mellitus. Int J Environ Res Public Health. (2021) 18(23):12612. 10.3390/ijerph182312612 34886337 PMC8656786

[13] Thomas C Jones H Whitworth-Turner C Louis J. High-intensity exercise in the evening does not disrupt sleep in endurance runners. Eur J Appl Physiol. (2020) 120(2):359–68. 10.1007/s00421-019-04280-w 31813044 PMC6989626

[14] Kolling S Wiewelhove T Raeder C Endler S Ferrauti A Meyer T Sleep monitoring of a six-day microcycle in strength and high-intensity training. Eur J Sport Sci. (2016) 16(5):507–15. 10.1080/17461391.2015.1041062 26062597

[15] Vesterinen V Häkkinen K Laine T Hynynen E Mikkola J Nummela A. Predictors of individual adaptation to high-volume or high-intensity endurance training in recreational endurance runners. Scand J Med Sci Sports. (2016) 26(8):885–93. 10.1111/sms.12530 26247789

[16] Vitale JA Bonato M Galasso L La Torre A Merati G Montaruli A Sleep quality and high intensity interval training at two different times of day: a crossover study on the influence of the chronotype in male collegiate soccer players. Chronobiol Int. (2017) 34(2):260–8. 10.1080/07420528.2016.1256301 27906554

[17] Frytz P Heib D Hoedlmoser K. Soccer, sleep, repeat: effects of training characteristics on sleep quantity and sleep architecture. Life-Basel. (2023) 13:8. 10.3390/life13081679 PMC10455405 37629536

[18] Ochoa-Lacar J Singh M Bird SP Charest J Huyghe T Calleja-Gonzalez J. How sleep affects recovery and performance in basketball: a systematic review. Brain Sci. (2022) 12:11. 10.3390/brainsci12111570 PMC9688068 36421894

[19] Sargent C Lastella M Halson SL Roach GD. How much sleep does an elite athlete need? Int J Sport Physiol Perform. (2021) 16(12):1746–57. 10.1123/ijspp.2020-0896 34021090

[20] Surała O Malczewska-Lenczowska J Sitkowski D Witek K Słomiński P Certa M Effect of training load on sleep parameters and biochemical fatigue markers in elite swimmers. Biol Sport. (2023) 40(4):1229–37. 10.5114/biolsport.2023.124843 37867745 PMC10588581

[21] Kong Z Zhang H Zhang M Jia X Yu J Feng J How to test the on-ice aerobic capacity of speed skaters? An on-ice incremental skating test for young skaters. Int J Environ Res Public Health. (2023) 20(4):2995. 10.3390/ijerph20042995 36833692 PMC9959978

[22] Hofman N Orie J Hoozemans MJM Foster C de Koning JJ. Wingate test as a strong predictor of 1500-m performance in elite speed skaters. Int J Sport Physiol Perform. (2017) 12(10):1288–92. 10.1123/ijspp.2016-0427 28253027

[23] Kuula L Pesonen AK. Heart rate variability and firstbeat method for detecting sleep stages in healthy young adults: feasibility study. Jmir Mhealth Uhealth. (2021) 9(2):e24704. 10.2196/24704 33533726 PMC7889416

[24] Troynikov O Watson CG Nawaz N. Sleep environments and sleep physiology: a review. J. Therm. Biol. (2018) 78:192–203. 10.1016/j.jtherbio.2018.09.012 30509635

[25] Roberts SSH Teo W Warmington SA. Effects of training and competition on the sleep of elite athletes: a systematic review and meta-analysis. Br J Sports Med. (2019) 53(8):513–22. 10.1136/bjsports-2018-099322 30217831

[26] Campbell EH Poudevigne M McFarlane S Dilworth L Irving R. Evidence that sleep is an indicator of overtraining during the competition phase of adolescent sprinters. J Sports Med (Hindawi Publ Corp). (2021) 2021:6694547. 10.1155/2021/6694547 33884272 PMC8041504

[27] Fullagar HH Skorski S Duffield R Hammes D Coutts AJ Meyer T. Sleep and athletic performance: the effects of sleep loss on exercise performance, and physiological and cognitive responses to exercise. Sports Med. (2015) 45(2):161–86. 10.1007/s40279-014-0260-0 25315456

[28] Thornton HR Duthie GM Pitchford NW Delaney JA Benton DT Dascombe BJ. Effects of a 2-week high-intensity training camp on sleep activity of professional rugby league athletes. Int J Sport Physiol Perform. (2017) 12(7):928–33. 10.1123/ijspp.2016-0414 27918662

[29] Yue T Liu X Gao Q Wang Y. Different intensities of evening exercise on sleep in healthy adults: a systematic review and network meta-analysis. Nat Sci Sleep. (2022) 14:2157–77. 10.2147/NSS.S388863 36540196 PMC9760070

[30] Gupta L Morgan K Gilchrist S. Does elite sport degrade sleep quality? A systematic review. Sports Med. (2017) 47(7):1317–33. 10.1007/s40279-016-0650-6 27900583 PMC5488138

[31] Min L Wang D You Y Fu Y Ma X. Effects of high-intensity interval training on sleep: a systematic review and meta-analysis. Int J Environ Res Public Health. (2021) 18(20):10973. 10.3390/ijerph182010973 34682718 PMC8535574

[32] Nummela A Hynynen E Kaikkonen P Rusko H. High-intensity endurance training increases nocturnal heart rate variability in sedentary participants. Biol Sport. (2016) 33(1):7–13. 10.5604/20831862.1180171 26985128 PMC4786581

[33] Nedelec M Halson S Abaidia AE Ahmaidi S Dupont G. Stress, sleep and recovery in elite soccer: a critical review of the literature. Sports Med. (2015) 45(10):1387–400. 10.1007/s40279-015-0358-z 26206724

[34] Hough J Leal D Scott G Taylor L Townsend D Gleeson M. Reliability of salivary cortisol and testosterone to a high-intensity cycling protocol to highlight overtraining. J Sports Sci. (2021) 39(18):2080–6. 10.1080/02640414.2021.1918362 33906585

[35] Haupt S Eckstein ML Wolf A Zimmer RT Wachsmuth NB Moser O. Eat, train, sleep—retreat? Hormonal interactions of intermittent fasting, exercise and circadian rhythm. Biomolecules. (2021) 11(4):516. 10.3390/biom11040516 33808424 PMC8065500

[36] Halson SL Juliff LE. Chapter 2—sleep, sport, and the brain. In: Wilson MR Walsh V Parkin B, editors. Progress in Brain Research. Amsterdam: Elsevier (2017). p. 13–31.: 234 (Reprinted). 10.1016/bs.pbr.2017.06.006 29031461

[37] Hamlin MJ Deuchrass RW Olsen PD Choukri MA Marshall HC Lizamore CA The effect of sleep quality and quantity on athlete’s health and perceived training quality. Front Sports Act Living. (2021) 3:705650. 10.3389/fspor.2021.705650 34568820 PMC8461238

[38] Leal DV Taylor L Hough J. Reproducibility of acute steroid hormone responses in men to short-duration running. Int J Sport Physiol Perform. (2019) 14(10):1430–7. 10.1123/ijspp.2018-1004 31034262

[39] Casado A Foster C Bakken M Tjelta LI. Does lactate-guided threshold interval training within a high-volume low-intensity approach represent the “next step” in the evolution of distance running training? Int J Environ Res Public Health. (2023) 20:5. 10.3390/ijerph20053782 PMC10000870 36900796

